# Protein comparison at the domain architecture level

**DOI:** 10.1186/1471-2105-10-S15-S5

**Published:** 2009-12-03

**Authors:** Byungwook Lee, Doheon Lee

**Affiliations:** 1 Korean BioInformation Center, KRIBB, Daejeon 305-806, Korea; 2 Department of Bio and Brain Engineering, KAIST, Daejeon 305-701, Korea

## Abstract

**Background:**

The general method used to determine the function of newly discovered proteins is to transfer annotations from well-characterized homologous proteins. The process of selecting homologous proteins can largely be classified into sequence-based and domain-based approaches. Domain-based methods have several advantages for identifying distant homology and homology among proteins with multiple domains, as compared to sequence-based methods. However, these methods are challenged by large families defined by 'promiscuous' (or 'mobile') domains.

**Results:**

Here we present a measure, called Weighed Domain Architecture Comparison (WDAC), of domain architecture similarity, which can be used to identify homolog of multidomain proteins. To distinguish these promiscuous domains from conventional protein domains, we assigned a weight score to Pfam domain extracted from RefSeq proteins, based on its abundance and versatility. To measure the similarity of two domain architectures, cosine similarity (a similarity measure used in information retrieval) is used. We combined sequence similarity with domain architecture comparisons to identify proteins belonging to the same domain architecture. Using human and nematode proteomes, we compared WDAC with an unweighted domain architecture method (DAC) to evaluate the effectiveness of domain weight scores. We found that WDAC is better at identifying homology among multidomain proteins.

**Conclusion:**

Our analysis indicates that considering domain weight scores in domain architecture comparisons improves protein homology identification. We developed a web-based server to allow users to compare their proteins with protein domain architectures.

## Background

Homology identification is part of a broad spectrum of genomic analyses, including the annotation of new whole genome sequences, the construction of comparative maps, the analysis of whole genome duplications and comparative approaches to identifying regulatory motifs [1]. The general method used to determine the function of newly discovered proteins is to transfer annotation from well-characterized homologous proteins sharing a common ancestry [2]. Current methods for the identification of homologous proteins can be largely classified into sequence-based and domain-based approaches [3]. Sequence comparison methods, such as BLAST and FASTA, are the commonly-used traditional approaches to identify homologous genes [4,5]. These methods assume that sequences with significant similarity share common ancestry, i.e. are homologs. However, the existence of multi-domain proteins and complex evolutionary mechanisms poses difficulties for sequence-based methods [6].

Domain-based methods use information of the domains contained in proteins [7]. Domains are the building blocks of all proteins, and present one of the most useful levels at which protein function can be understood [8]. Although the concept of a 'domain' now permeates biological descriptions, there are several definitions directed at different levels of the protein [9]. In structural biology, a domain is defined as a spatially distinct, compact and stable protein structural unit that could conceivably fold and function in isolation. Domains are also defined as distinct regions of protein sequence that are highly conserved throughout evolution. These are described as sequence homologs and are often present in different molecular contexts. Sequence-based domain definitions represent one of the most convenient and practically important levels at which the evolution and function of both proteins and domains can be understood.

Domain-based approaches identify homologous proteins generally by comparing protein domain architecture, which is the linear order of the individual domains in multidomain protein. About two thirds of proteins in prokaryotes and 80% of proteins in eukaryotes are multi-domain proteins [10]. Studies of domain-based methods indicate that comparing domain architecture is a useful method for classifying evolutionarily related proteins and detecting evolutionarily distant homologs [11]. Several studies have proposed tools for domain architecture comparison, such as CDART [12] and PDART [9]. However, these methods are challenged by large families defined by 'promiscuous' (or 'mobile') domains, which combine in many ways with other domains to form different proteins [13]. Promiscuous domains have typically auxiliary functions to the primary role of protein, acting as signal transducers, or adapters [14,15]. They also play a major role in creating diversity of protein domain architecture in the proteome [16]. Because they are not directly related by homology, they should be given less importance in homology identification than key domains. Another problem inherent to these domain-based measures is that they treat all proteins in a domain architecture equally. They cannot discriminate among proteins belonging to the same domain architecture. Since most domain architectures consist of many proteins, identification methods are needed to determine which protein is the most homologous to the query protein within a set of proteins belonging to the same domain architecture.

Here we present a measure, called Weighed Domain Architecture Comparison (WDAC), of domain architecture similarity, which can be used to identify homologs of multidomain proteins. The key ideas are the use of weight scores for domain promiscuity and combining domain architecture comparison with sequence similarity method. The weight scores are calculated based on a domain's frequency and versatility in RefSeq [17] proteins. The effectiveness of our method is evaluated using human and nematode proteomes. We developed a web-based server to allow users to compare their proteins with protein domain architectures. The server is available at http://wdac.kr/.

## Methods

### Domain assignment

In this study we used the Pfam [18] database to analyze the domain organization of proteins. Pfam is a large and widely used database of protein domains and families. Pfam contains curated multiple sequence alignments for each family, as well as profile hidden Markov models for finding these domains in new sequences. Pfam also provides better genomic coverage than structure-based domain assignments, such as CATH [19] and SCOP [20], particularly for membrane proteins.

### Measuring the strength of domain promiscuity

To measure the strength of domain promiscuity, we considered two features of protein domains, the first of which is domain abundance. Compared to non-promiscuous domains, promiscuous domains appear in many proteins because they are needed to perform auxiliary functions. Vogel et al. [21] have shown that the combination tendencies of domains can be explained by a random evolutionary process model, in which a highly abundant domain tends to form more combinations. To measure the abundance of a domain, we defined the Inverse Abundance Frequency (IAF). The basic concept of IAF is derived from the Inverse Document Frequency (IDF), a statistic commonly used in information retrieval. IDF is a measure based on the observation that a word that occurs in very few documents is more likely to differentiate between subjects than a word that occurs frequently [22]. Namely, IDF is a measure of the general importance of a term. The IDF score is obtained by dividing the number of all documents by the number of documents containing the term, and then taking the logarithm of that quotient. For example, if 'cow' appears in 100 documents out of a total of 10,000 and 'bovine' in 10 documents, the IDF scores of 'cow' and 'bovine' are 0.2 and 0.1, respectively. Thus, the word 'cow' conveys less information about the subject of the document than the word 'bovine'. The number of documents containing a term and the number of documents in the corpus are analogous to the proteins containing a domain and the total number of proteins under study in the IAF statistic, respectively. The definition of IAF for a domain, *d*, is

where *p*_*t *_is the number of total proteins and *p*_*d *_is the number of proteins containing domain *d*.

**Figure 1:**
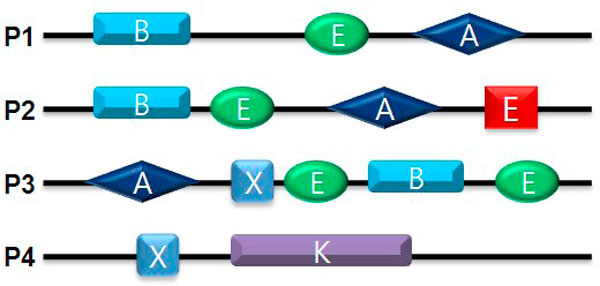
**Example calculation of the weight scores of domains**. Black lines represent protein sequences and the colored boxes, circles, and diamonds represent different domains. The number of occurrences of two exemplary domains, A and B, is three (P1, P2, and P3), and the number of distinct partner domains of A and B is three (E, D, and A) and one (E), respectively. The IAF scores of A and B are 0.125, and the IV scores of A and B are 0.3 and 1.0, respectively. We can obtain the final weight score of domain A (0.038) and domain B (0.125) from the IDF and IV scores. From the weight scores of the two domains, we can determine that domain A is a more promiscuous domain than domain B.

The second feature of protein domains that we consider is domain versatility. Promiscuous domains occurring in many protein clusters have many partner domain families while highly conserved domains appear in a small number of protein clusters and their neighbor domains are also conserved during evolution [16]. Thus, domains with the same abundance could have a different number of distinct partner domain families. To measure the versatility of a domain, we defined the Inverse Versatility (IV) obtained from the inverse of the number of distinct partner domain families at the N- and C-sides adjacent to a domain. The definition of the IV of a domain, *d*, is

where *f*_*d *_is the number of distinct domain families adjacent to domain *d*. The weight score of a domain is simply calculated by the product of the IDF and the IV of a domain. Let us consider the theoretical example (Figure 1), where both domain A and domain B occur three times. Domain A has four distinct neighbors and domain B has only one distinct neighbor. Since the weight score of domain A is lower than that of domain B, domain A is more promiscuous than domain B.

### Comparison of domain architectures using weight scores

Using the domain weight scores, we compared domain architectures. First, the shared distinct domain families are compared. We represented the two sets of domains derived from two architectures as the indices, which are built using the vector-space model (VSM) [23]. Domain architectures were converted into a vector in which each component corresponds to the weight score of a domain. The similarity of the two vectors is measured by determining their cosine similarity, a measure based on the angle between two vectors (commonly used in text mining algorithms). If *x *and *y *are vectors of two domain architectures *X *and *Y*, the cosine similarity is defined

The range of the cosine similarity is [0, 1], where 1 indicates that *x *and *y *have the same domains and 0 indicates that they share no domains.

Second, domain orders were considered. To measure the order similarity, we compared shared domain pairs between two domain architectures. In domain evolution, two- or three-domain combinations, called supradomain, are re-used in different protein context, and domain pairs in protein domain architecture occur in only one order, with only about 2% of such pairs occurring on both possible orders. The order similarity is measured by dividing the shared domain pairs (*Qs*) by the total domain pairs (*Qt*). The function is defined by

The final similarity score between two domain architectures, *X *and *Y*, is obtained by combining the indices from equations 3 and 4 (each normalized to [0, 1]) using a simple linear function.

### Pipeline for domain architecture comparison

**Figure 2:**
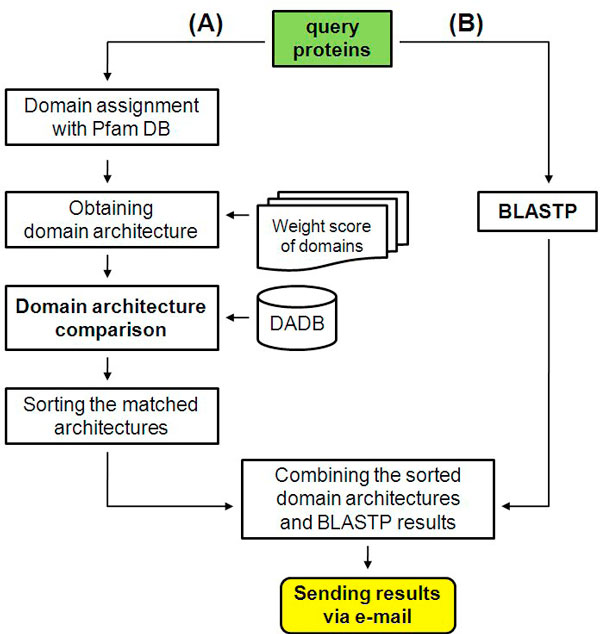
**Workflow for the identification of protein homology**. The pipeline combines sequence similarity information and domain architecture comparison methods.

We constructed an automatic pipeline for identifying homologs of proteins (Figure 2). The pipeline programs were written in Perl and consist of four main steps. First, the pipeline assigns Pfam domains to a query protein and extracts a domain architecture from the Pfam annotation. Second, the query domain architectures are compared against the domain architecture database. Third, the query proteins are compared with RefSeq proteins using BLASTP [24]. Lastly, matched domain architectures and BLAST results are combined and sorted according to their similarity scores.

### Web-based server

We developed a web-based server to provide a back-end pipeline for protein homology and to allow users to compare their protein sequences with a domain architecture database. The web interface is implemented with static HTML and CGI scripts, and MySQL DBMS is used to store the database.

## Results and discussion

### Obtaining weight scores of protein domains

We downloaded 6,042,750 protein sequences from the RefSeq database (Release 32). The domain content of the sequences was analyzed with Pfam 23.0 containing 10,340 families. The Pfam domain annotations of all RefSeq proteins were obtained from the Similarity Matrix of Proteins (SIMAP) [25] database. We filtered domain hits in proteins with a cutoff E-value of 0.01 and excluded proteins without Pfam signatures. We extracted all the Pfam domains from the Pfam-annotated proteins

**Figure 3:**
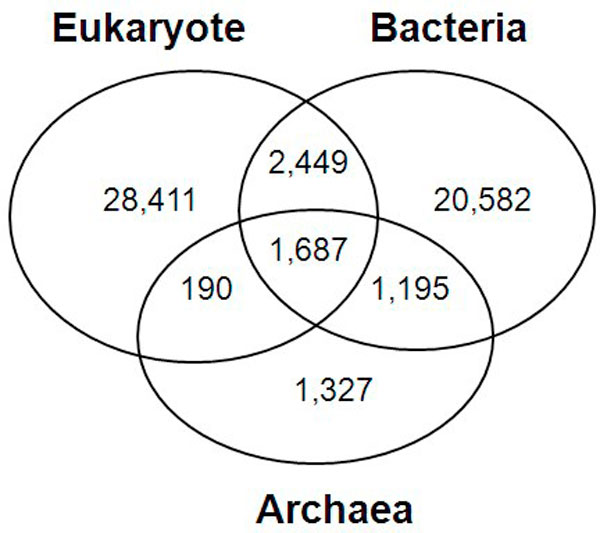
**The distribution of domain architectures across the three kingdoms of life**. Ninety percent of the domain architectures are kingdom-specific.

Of the 6,042,750 RefSeq proteins, 3,942,678 (65%) contain more than one Pfam domain. These Pfam-annotated proteins were converted into domain architectures, in which we obtained 55,841 distinct domain architectures. The domain architecture data show that 90% of the domain architectures are kingdom-specific. Thus, we classified the 55,841 domain architectures into three kingdoms: Eukaryote, Bacteria, and Archaea (Figure 3). From these domain architectures, we extracted 8,775 domains and then divided them into the three kingdoms, where 17% of all the domains are common to all three kingdoms of life whereas 54% appear only in one kingdom.

Because domains are differently distributed in the three kingdoms and some domains are absent or present in one or two kingdoms, we assigned three kingdom-specific weight scores to each domain based on its abundance and versatility in the three kingdoms. To measure domain abundance, we obtained the kingdom-specific protein frequency for each domain. Most domains occur in a hundred or fewer proteins, but a few domains are highly duplicated and occur in over 10,000 sequences. The most abundant domain in the three kingdoms is ABC_tran (PF00005), appearing in 54,980 bacterial proteins. To measure domain versatility, we obtained kingdom-specific N- and C-side distinct domain families adjacent to each domain. We found that most domains have one or two distinct adjacent domain families. The features of the obtained domain versatility are consistent with earlier reports that the number of different partner domains for a single domain or for a domain combination follows a power law distribution: many domains or domain combinations have only very few different N-terminal or C-terminal partner domains. The most versatile domain in the three kingdoms is Ank (PF00023), having 220 distinct partner domain families in eukaryotes.

We calculated kingdom-specific IDF and IV scores for all domains using eq. 1 and 2, and obtained weight scores for each domain by the product of the IAF and IV scores. The scores were multiplied by 10 to facilitate computation. These domain's scores represent their importance in the protein universe and are used in the comparison of domain architectures. The analysis of the weight scores indicates that they are distributed 0.2 to 138.00 (Table 1), where most scores are over 100 and a small number of domains have scores below 20. Top ten domains with lower scores in the three Kingdoms are given in Table 2 and the weight scores distribution over all Pfam domains is given at the website.

**Table 1:** The distribution of weight scores across the three kingdoms of life.

Weight scores	Eukaryote	Bacteria	Archaea	Total
120 - 140	3,767	4,430	-	8,197
100 - 120	1,172	1,111	6,728	9,011
80 - 100	1,229	971	825	3,025
60 - 80	66	259	666	991
40 - 60	820	441	104	1,365
20 - 40	741	628	282	1,651
0 - 20	980	935	170	2,085

**Table 2:** Top-ten promiscuous domains in the three Kingdoms Numbers in parenthesis is the weight scores of Pfam domains.

Order	Eukaryote	Bacteria	Archaea
1	Ank (0.19)	TPR_2 (0.41)	Fer4 (0.86)
2	WD40 (0.24)	Response_reg (0.45)	PKD (1.71)
3	zf-C2H2 (0.3)	ABC_tran (0.47)	CBS (1.82)
4	zf-C3HC4 (0.3)	Acetyltransf_1 (0.50)	Radical_SAM (2.15)
5	RRM_1 (0.41)	Fer4 (0.62)	AAA (2.50)
6	7tm_1 (0.44)	TPR_1 (0.63)	Response_reg (2.79)
7	PH (0.46)	HATPase_c (0.64)	HATPase_c (2.81)
8	efhand (0.46)	fn3 (0.73)	HTH_5 (2.84)
9	EGF (0.48)	HTH_3 (0.74)	PAS (3.08)
10	MFS_1 (0.53)	HisKA (0.75)	TPR_2 (3.15)

**Figure 4:**
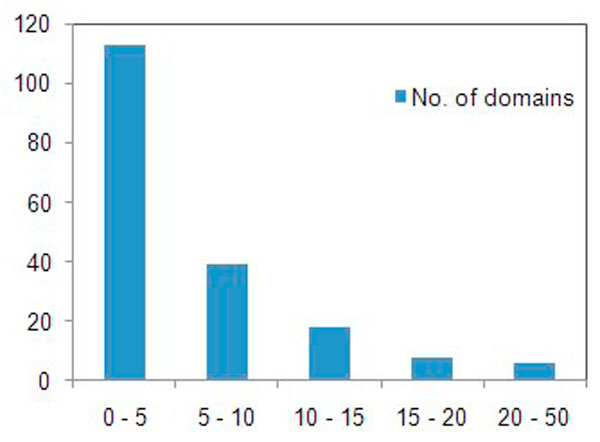
**The distribution of weight scores of 215 eukaryotic promiscuous domains**. All of the known promiscuous domains have very low weight scores, 152 (83%) mostly below 10. It means that the calculated weight scores represent domain promiscuity and importance of protein domains.

We examined the weight scores of previously known promiscuous domains to identify relationship between weight scores and domain promiscuity. To do this, 215 eukaryotic promiscuous domains published by Basu et al. [14] were used. These promiscuous domains consist of 76 Pfam domains and 139 Smart domains, and are involved in protein-protein interaction and have crucial roles interaction networks. To facilitate comparison between these known promiscuous domains and the weight scores, we converted the 139 Smart domains into the corresponding Pfam domains, where 108 Smart domains could be converted. We found that all of the known promiscuous domains have very low weight scores, 152 (83%) mostly below 10 (Figure 4). It means that the calculated weight scores represent domain promiscuity and importance of protein domains.

### Performance evaluation

To assess the effect of domain weight scores on domain architecture comparison, the WDAC (weighted method) was compared to the general unweighted domain architecture comparison (DAC) method using all complete *Homo sapiens *(human) and *Caenorhabditis elegans *(nematode) protein sequences. In the DAC method, domain weight scores are not considered. To implement the DAC method, we used Jaccard similarity [26], which is commonly used in information retrieval, instead of the measure of cosine similarity used in the WDAC method. The Jaccard similarity can be calculated by the following equation:

where *f*_11 _is the number of domains common to both sequences X and Y, *f*_10 _is the number of domains in X, and *f*_01 _is the number of domains in Y.

We extracted all complete human and nematode protein sequences from RefSeq proteins, yielding 32,999 human and 23,220 nematode protein sequences. Among these proteins, 23,295 human and 14,522 nematode proteins have detectable Pfam domain information. Among the human proteins, we selected 9,764 proteins that contain more than 2 Pfam domains and performed domain architecture comparisons between the selected human proteins (≥ two domains) and those from the nematode proteome (≥ one domain) using the WDAC and DAC algorithms.

To validate homologous pairs of human and nematode proteins, we used the HomoloGene database [27], a NCBI dataset that curates sets of orthologs from the annotated genes of several completely sequenced eukaryotic genomes. Among the 44,481 groups in HomoloGene release 61, we selected 2,559 groups that have both the selected human proteins and nematode proteins. From the comparison results, we extracted the WDAC and DAC results that have the same HomoloGene ID in the query (human) and the best matched protein (nematode). The results show that the number of true positive values in the WDAC and DAC results are 2,328 (91%) and 2,175 (85%) respectively, which means that considering weight scores in domain architecture comparison can improve homology identification.

**Figure 5:**
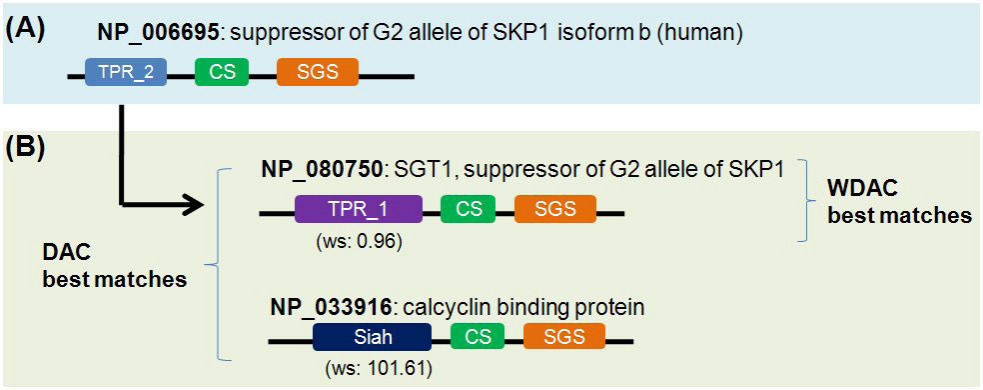
**The best matches of the WDAC and DAC results for a human protein, NP_006695**. (A) Query protein (human). (B) The best-matched proteins in the WDAC and DAC results. DAC cannot distinguish between two proteins (NP_080750 and NP_033916), while WDAC can identify more homologous proteins by using weight scores.

In addition, we found that the WDAC results have more specific homologs than the DAC results. Figure 5 shows the query results of a human protein NP_006695 (suppressor of the G2 allele of SKP1 isoform b). The best matched protein from the WDAC results is NP_080750 (SGT1, suppressor of the G2 allele of SKP1), while DAC results have two proteins, NP_080750 and NP_033916, as the best matched protein. The reason that DAC cannot discriminate between the two proteins is that DAC treats two domains, TPR_1 and Siah, equally.

### Construction of web server

**Figure 6:**
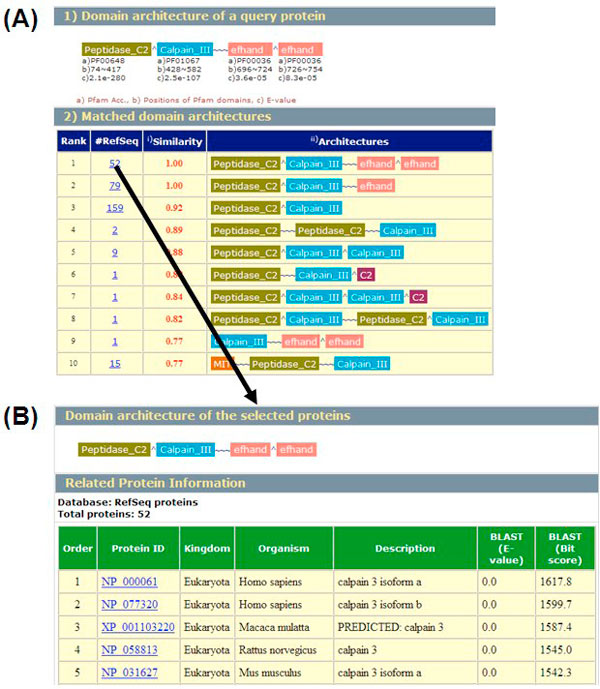
**Screenshot of the results of the WDAC server for a query protein**. (A) Matched domain architecture. (B) List of protein belonging to the selected domain architecture.

The query interface accepts protein sequences in FASTA format, and the maximum number of input protein sequences for a single submission is 100 and the length of each sequence is limited to 5000 residues. The output of the server is an HTML-formatted file, which consists of three parts: query domain architecture with Pfam domains, matched domain architecture, and domain information (Figure 6). For more than two sequences, users must input an email address to receive the WDAC results.

## Conclusion

There are several current homology methods which compare domain architectures. However, these methods are challenged by large families defined by promiscuous domains. To cope with the promiscuous domain problem, we present a method for measuring the similarity among protein domain architectures based on their Pfam-A domain annotations. The Pfam database may contain a small number of false positives and false negatives. Nevertheless, it is currently one of the most useful and practical domain annotation databases for protein sequences. In this study, we consider domain weight scores, obtained based on the abundance and versatility of domains. Our analysis indicates that considering domain weight scores in domain architecture comparison improves the performance of protein homology identification. The WDAC algorithm is also effective in resolving some issues that have baffled traditional sequence-based comparison methods, such as the comparison of proteins with promiscuous domain(s). The WDAC algorithm and its web server could be used to explore the underlying evolutionary relationships among proteins at the level of their whole domain architectures, rather than at the single-domain or protein sequence level.

## Competing interests

The authors declare that they have no competing interests.

## Authors' contributions

BL designed the algorithm, carried out the programming, analyzed the results and wrote the manuscript. DL directed the entire study. All authors read and approved the final manuscript.

## Note

Other papers from the meeting have been published as part of *BMC Genomics *Volume 10 Supplement 3, 2009: Eighth International Conference on Bioinformatics (InCoB2009): Computational Biology, available online at http://www.biomedcentral.com/1471-2164/10?issue=S3.

## References

[B1] Song N Sedgewick RD Durand D Domain architecture comparison for multidomain homology identification J Comput Biol 2007 14 4 496 516 10.1089/cmb.2007.A009 17572026

[B2] Punta M Ofran Y The rough guide to in silico function prediction, or how to use sequence and structure information to predict protein function PLoS Comput Biol 2008 4 10 e1000160 10.1371/journal.pcbi.1000160 18974821 PMC2518264

[B3] Ponting CP Russell RR The natural history of protein domains Annu Rev Biophys Biomol Struct 2002 31 45 71 10.1146/annurev.biophys.31.082901.134314 11988462

[B4] Lee B Hong T Byun SJ Woo T Choi YJ ESTpass: a web-based server for processing and annotating expressed sequence tag (EST) sequences Nucleic acids research 2007 35 Web Server W159 162 10.1093/nar/gkm369 17526512 PMC1933161

[B5] Lee B Shin G CleanEST: a database of cleansed EST libraries Nucleic acids research 2009 37 Database D686 689 10.1093/nar/gkn648 18832365 PMC2686460

[B6] Song N Joseph JM Davis GB Durand D Sequence similarity network reveals common ancestry of multidomain proteins PLoS Comput Biol 2008 4 4 e1000063 10.1371/journal.pcbi.1000063 18475320 PMC2377100

[B7] Hollich V Sonnhammer EL PfamAlyzer: domain-centric homology search Bioinformatics (Oxford, England) 2007 23 24 3382 3383 10.1093/bioinformatics/btm521 17977882

[B8] Chothia C Gough J Vogel C Teichmann SA Evolution of the protein repertoire Science 2003 300 5626 1701 1703 10.1126/science.1085371 12805536

[B9] Lin K Zhu L Zhang DY An initial strategy for comparing proteins at the domain architecture level Bioinformatics (Oxford, England) 2006 22 17 2081 2086 10.1093/bioinformatics/btl366 16837531

[B10] Tordai H Nagy A Farkas K Banyai L Patthy L Modules, multidomain proteins and organismic complexity The FEBS journal 2005 272 19 5064 5078 10.1111/j.1742-4658.2005.04917.x 16176277

[B11] Fong JH Geer LY Panchenko AR Bryant SH Modeling the evolution of protein domain architectures using maximum parsimony Journal of molecular biology 2007 366 1 307 315 10.1016/j.jmb.2006.11.017 17166515 PMC1858635

[B12] Geer LY Domrachev M Lipman DJ Bryant SH CDART: protein homology by domain architecture Genome research 2002 12 10 1619 1623 10.1101/gr.278202 12368255 PMC187533

[B13] Bjorklund AK Ekman D Light S Frey-Skott J Elofsson A Domain rearrangements in protein evolution Journal of molecular biology 2005 353 4 911 923 10.1016/j.jmb.2005.08.067 16198373

[B14] Basu MK Carmel L Rogozin IB Koonin EV Evolution of protein domain promiscuity in eukaryotes Genome research 2008 18 3 449 461 10.1101/gr.6943508 18230802 PMC2259109

[B15] Lee B Lee D DAhunter: a web-based server that identifies homologous proteins by comparing domain architecture Nucleic Acids Res 2008 36 Web Server W60 64 10.1093/nar/gkn172 18411203 PMC2447808

[B16] Basu MK Poliakov E Rogozin IB Domain mobility in proteins: functional and evolutionary implications Brief Bioinform 2009 10 3 205 216 10.1093/bib/bbn057 19151098 PMC2722818

[B17] Pruitt KD Tatusova T Maglott DR NCBI reference sequences (RefSeq): a curated non-redundant sequence database of genomes, transcripts and proteins Nucleic acids research 2007 35 Database D61 65 10.1093/nar/gkl842 17130148 PMC1716718

[B18] Finn RD Tate J Mistry J Coggill PC Sammut SJ Hotz HR Ceric G Forslund K Eddy SR Sonnhammer EL The Pfam protein families database Nucleic acids research 2008 36 Database D281 288 18039703 10.1093/nar/gkm960 PMC2238907

[B19] Greene LH Lewis TE Addou S Cuff A Dallman T Dibley M Redfern O Pearl F Nambudiry R Reid A The CATH domain structure database: new protocols and classification levels give a more comprehensive resource for exploring evolution Nucleic acids research 2007 35 Database D291 297 10.1093/nar/gkl959 17135200 PMC1751535

[B20] Andreeva A Howorth D Chandonia JM Brenner SE Hubbard TJ Chothia C Murzin AG Data growth and its impact on the SCOP database: new developments Nucleic acids research 2008 36 Database D419 425 18000004 10.1093/nar/gkm993 PMC2238974

[B21] Vogel C Teichmann SA Pereira-Leal J The relationship between domain duplication and recombination Journal of molecular biology 2005 346 1 355 365 10.1016/j.jmb.2004.11.050 15663950

[B22] Yu S Van Vooren S Tranchevent LC De Moor B Moreau Y Comparison of vocabularies, representations and ranking algorithms for gene prioritization by text mining Bioinformatics (Oxford, England) 2008 24 16 i119 125 10.1093/bioinformatics/btn291 18689812

[B23] Glenisson P Coessens B Van Vooren S Mathys J Moreau Y De Moor B TXTGate: profiling gene groups with text-based information Genome Biol 2004 5 6 R43 10.1186/gb-2004-5-6-r43 15186494 PMC463076

[B24] Altschul SF Madden TL Schaffer AA Zhang J Zhang Z Miller W Lipman DJ Gapped BLAST and PSI-BLAST: a new generation of protein database search programs Nucleic Acids Res 1997 25 17 3389 3402 10.1093/nar/25.17.3389 9254694 PMC146917

[B25] Rattei T Tischler P Arnold R Hamberger F Krebs J Krumsiek J Wachinger B Stumpflen V Mewes W SIMAP--structuring the network of protein similarities Nucleic Acids Res 2008 36 Database D289 292 18037617 10.1093/nar/gkm963 PMC2238827

[B26] Balestre M Von Pinho RG Souza JC Lima JL Comparison of maize similarity and dissimilarity genetic coefficients based on microsatellite markers Genet Mol Res 2008 7 3 695 705 10.4238/vol7-3gmr458 18752197

[B27] Sayers EW Barrett T Benson DA Bryant SH Canese K Chetvernin V Church DM DiCuccio M Edgar R Federhen S Database resources of the National Center for Biotechnology Information Nucleic Acids Res 2009 37 Database D5 15 10.1093/nar/gkn741 18940862 PMC2686545

